# The Effectiveness of the Flipped Classroom Approach Over Traditional Teaching for a Comprehensive Understanding of Embryology by the First-Year MBBS Professionals

**DOI:** 10.7759/cureus.88842

**Published:** 2025-07-27

**Authors:** Amit K Shreevastava, Rajat S Das, Balkund Kailash

**Affiliations:** 1 Anatomy, All India Institute of Medical Sciences Raebareli, Raebareli, IND

**Keywords:** competency-based medical education, embryology, flipped classroom, medical undergraduates, traditional teaching

## Abstract

Introduction

This study compared the effectiveness of the flipped classroom model with traditional teaching methods in improving embryology understanding among first-year MBBS students in competency-based medical education. The goal was to foster a deep understanding of the subject and produce well-rounded medical professionals who can contribute to the healthcare sector.

Materials and methods

The study involved first-year medical undergraduates. Traditional teaching was used for five months initially, followed by the flipped classroom approach for the subsequent five months. Post-tests followed each session, with a Likert scale feedback survey involving students and faculty in the anatomy department yielding insightful results.

Results

Post-test scores in the flipped classroom (6.54 ± 0.87) significantly surpassed those of traditional teaching (5.94 ± 0.78) (P = 0.001). Chi-square testing revealed a noteworthy difference in post-test scores category favoring the flipped classroom (P = 0.002). Additionally, cognitive assessment demonstrated the flipped classroom's superiority in knowledge, application, and analysis (P = 0.01, P = 0.01, P = 0.003, respectively). Feedback indicated the flipped classroom's efficacy in enhancing competency, with students excelling in embryology topics. Overall, students and faculty members perceive integrating the flipped classroom into the curriculum positively. This strengthens the fact that the model is more effective than traditional teaching methods in improving embryology comprehension among first-year MBBS students.

Conclusion

The findings suggest that implementing this method can improve students' knowledge, application, and analytical skills in every subject. These results have positive implications for medical education, reinforcing knowledge acquisition with far better understanding and thus enhancing the analytical skills for efficient application.

## Introduction

Various newer teaching-learning models are being inducted to upgrade medical education to meet the upcoming challenges of the changing healthcare environment and to produce medical graduates who are globally relevant to provide effective and efficient healthcare facilities to the communities [1-5]. Flipped classroom (FC) teaching is one of the growing pedagogical teaching-learning tools increasingly employed in the current competency-based medical education [1,2]. Compared to the passive teacher-centered traditional teaching (TT) approach, the FC is a student-centric active blended learning approach using online and face-to-face learning activities [1-6]. In FC, students obtain basic knowledge through study materials (short videos, lecture PPTs, web links for related topics, handouts, etc.) given before class by the concerned faculty member and utilize this knowledge to develop higher-order learning skills during class [1-6]. The primary focus of the class session is to foster active learning processes by engaging students in problem-solving or case-based scenarios, allowing them to apply and demonstrate their acquired knowledge [1-6]. We live in an era of advanced technology, and the medical field has embraced this innovation to enhance learning. The Flipped Classroom (FC) exemplifies a teaching technique that effectively utilizes the latest technologies. This approach ensures that instructors are actively engaged during sessions, addressing any questions that arise about the material and helping students reach higher levels of Bloom's taxonomy [7]. The goal is to successfully achieve the desired competencies for the embryology course in the first year of the professional MBBS program.

Embryology is a fundamental anatomical discipline that deals with the intricate development of various organs and biological systems of the human body [8-10]. The study of human embryology is very dynamic and full of fascination [11]. It deals with both normal and abnormal human developmental mechanisms [8-10]. It has significant practical potential and serves as the perfect link between basic sciences and clinical specializations [8-11]. Undeniably, a sound knowledge of embryology in the early stages of undergraduate education will provide a strong logical foundation for comprehending the developmental basis of pediatric and adult diseases [8-11]. This will help them draw accurate conclusions during clinical courses. This strong foundation of embryology subject facilitates the health professional to guide, counsel, and treat the patient in various complicated medical issues like infertility, in vitro fertilization, use of contraception, congenital birth defect, prenatal development, cryopreservation of embryos, surrogacy, stem cells, cloning, etc. [8-11]. The majority of medical colleges in India use traditional teaching (TT) methods to cover embryology topics. In the TT method, undergraduate medical students learn the topics through PowerPoint presentations, chalk and board, 2D models, and diagrams [2,8]. Faculties often find it challenging to teach embryology using these traditional techniques. Similarly, students also find it difficult to learn embryology through these methods, resulting in a progressive lack of interest. Consequently, the students perform below par in the subject during the assessment [11].

Although students can gain essential knowledge through TT methods, gaining a thorough understanding, improved application, development of an analytical mindset, and a creative approach requires a clear spatial visualization of the subject. This visualization not only aids with orientation but also ensures a strong comprehension of the topic [7-11]. To achieve the perfect spatial visualization of the subject, we also need a technological-friendly teaching-learning method that helps in achieving the desired goal [7,8,11-13]. The FC is the ultimate approach to getting the desired result [7,12,13]. Therefore, to effectively imbibe the subject of embryology, students need a more dynamic teaching-learning approach. For this purpose, we have selected a combination of embryology topics and employed FC for the study. This will help students develop better concepts and become competent medical graduates [5].

The rationale for choosing the flipped classroom approach and focusing on embryology topics stemmed from their relevance in the medical curriculum. With the rapid evolution of the field of medical science, traditional teaching methodologies pose challenges for medical students in achieving a comprehensive understanding of subjects within a limited timeframe. The integration of ideal technology, such as the FC model, has the potential to revolutionize medical education and enhance learning productivity and engagement. This transition can lead to the development of competent medical professionals who can effectively manage healthcare systems. Given the dearth of prior comprehensive research on this topic, we must provide easily understandable and beneficial information for MBBS students to support their future careers. Consequently, we undertook this study.

The study aimed to achieve several specific objectives: to evaluate the effectiveness of the FC teaching approach in the anatomy and embryology course, to compare the effectiveness of the FC with TT methods, and to gather insights from both students and faculty regarding their perceptions of implementing the FC in competency-based embryology courses.

## Materials and methods

The interventional study was conducted after obtaining ethical approval from the Institutional Ethics Committee of All India Institute of Medical Sciences (AIIMS), Raebareli, India. The approval number was F. 3/BIOETHICS/AIIMS-RBL/APPRO/IM/2021/2023-3 dated 22.02.2022.

Inclusion criteria

First-year MBBS students (above 18 years) of the institute have been chosen for the study after taking their written informed consent. The language of the survey was English.

Exclusion criteria

Students who were not willing to participate in the study.

Study description

**Figure 1:**
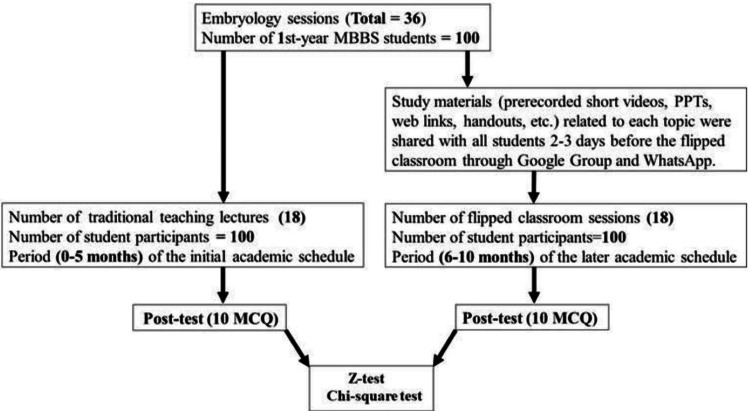
Illustration of the study design for embryology sessions (Anatomy) for first-year MBBS students. MCQ = Multiple choice questions; Total number of questions = 10; Total marks = 10; Number of student participants = 100.

A total of one hundred first-year MBBS students participated in a study on Embryology after providing written informed consent. The study included 36 lectures, divided equally between the TT method and the FC approach, conducted over 10 months. In the first five months, lectures were delivered using traditional methods, followed by the FC approach for the next five months. For the FC sessions, faculty provided materials - such as pre-recorded videos, PowerPoint presentations, and article links - at least 2-3 days before class via a shared Google group and WhatsApp, allowing students time to prepare. Post-tests with 10 multiple-choice questions were administered after each session for both methods (Figure 1).

To prevent bias, post-test questions and their assessment, covering various domains of Bloom’s taxonomy, along with the survey questions (Appendices 1 & 2), were prevalidated by other faculty members (four in number) of the department. The questionnaires were based on a five-point Likert Scale - strongly agree, agree, neutral, disagree, and strongly disagree (Appendices 1 & 2). The questionnaire's internal consistency was assessed using Cronbach’s alpha test for reliability. The reliability coefficient for all the items on the feedback questionnaire was 0.83. The feedback from students and faculty members in the Anatomy department was collected anonymously.

In the TT method, the topics covered included general embryology, the axial skeleton, the muscular system, the limbs, the integumentary system, and the respiratory system. In the FC approach, the topics included the cardiovascular system, the digestive system, the head and neck, the urogenital system, the central nervous system, the ear, the eye, and molecular regulation and signaling.

Subject matter experts in the Anatomy department confirmed that the distribution of topics was balanced across both halves of the course. By having the same group of students participate in both halves, we ensured consistency among the participants, which allowed for a valid comparison of the baseline groups. This approach minimized the impact of confounding variables.

Statistical analysis

All the quantitative and qualitative data was recorded in a Microsoft Excel 2019 spreadsheet (Microsoft Corp., Redmond, WA, USA). The spreadsheet was utilized to create tables and graphical charts. The statistical analyses were performed using the Microsoft Excel Analysis ToolPak. We conducted a descriptive statistical analysis to assess the normality of the post-test score data (skewness and kurtosis). A Chi-square (χ2) test was used for categorical variables, while a Z test was utilized for continuous variables. The measured data was depicted as mean ± standard deviation. A p-value ≤ 0.05 was considered to be significant.

## Results

Considering the guideline, when skewness and kurtosis are close to zero, the pattern of responses is expected to follow a normal distribution [14]. We conducted a descriptive statistical analysis of the post-test score data to assess the distribution's normality. The skewness and kurtosis of the FC and the TT were determined to be -0.1 and -0.5, and -0.04 and -0.06, respectively (Table 1).

**Table 1:** Descriptive statistical analysis of the post-test scores in the flipped classroom and traditional teaching respectively. Total marks = 10; Total number of student participants = 100.

Descriptive statistical analysis	Flipped Classroom	Traditional Teaching
Mean	6.543104575	5.944444444
Standard Error	0.086571035	0.077705594
Median	6.583333333	6
Mode	6.666666667	5.722222222
Standard Deviation	0.865710349	0.777055942
Sample Variance	0.749454409	0.603815937
Kurtosis	-0.521003116	-0.063863166
Skewness	-0.117861548	-0.043505718
Range	3.666666667	3.5
Minimum	4.666666667	4.111111111
Maximum	8.333333333	7.611111111
Sum	654.3104575	594.4444444
Count	100	100
Confidence Level (95.0%)	0.171775715	0.154184757

**Figure 2:**
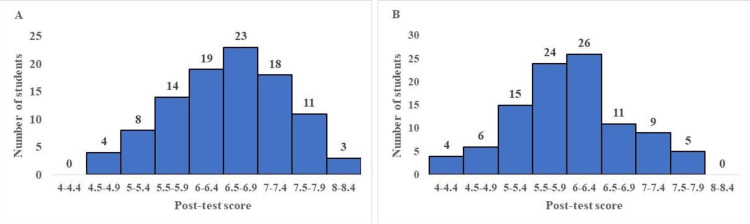
A Histogram chart showing the distribution of students in the flipped classroom method (A) and the traditional teaching method (B) across each post-test score range. Total marks = 10; Number of student participants = 100.

In our study, the post-test score data appears to be distributed normally as shown in Figure 2a and Figure 2b (Figure 2a for FC and Figure 2b for TT). The number of students present for each post-test score range is shown in Table 2.

**Table 2:** The number of students in the flipped classroom and the traditional teaching for each post-test score range, respectively. Total marks = 10; Total number of student participants = 100.

Post-test scores range	Number of students (Flipped Classroom)	Number of students (Traditional Teaching)
4-4.4	0	4
4.5-4.9	4	6
5-5.4	8	15
5.5-5.9	14	24
6-6.4	19	26
6.5-6.9	23	11
7-7.4	18	9
7.5-7.9	11	5
8-8.4	3	0
Total	100	100

**Figure 3:**
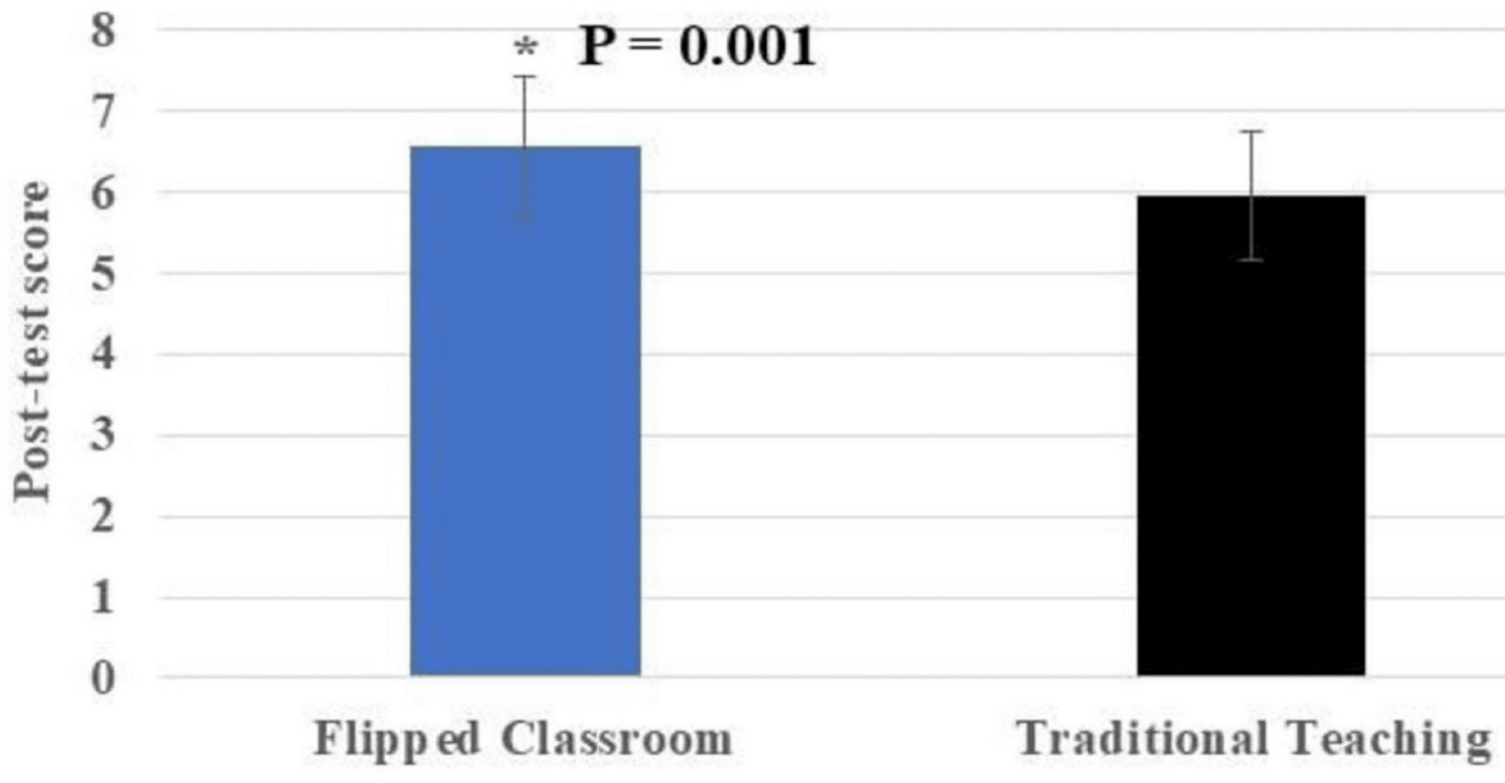
Mean post-test scores compared between the flipped classroom (6.54±0.87) and traditional teaching (5.94±0.78) using 2D clustered columns. Error bars show standard deviation. * Indicates statistical significance (P ≤ 0.05). Total marks = 10; Total number of student participants = 100.

When comparing the TT method (5.94 ± 0.78) with the FC method (6.54 ± 0.87), the FC method resulted in a higher mean post-test score for students, as shown in Table 3. To test for statistical significance between the FC and TT groups, the Z-test: two samples for Means (Table 3) was employed. The results (Table 3, Figure 3) showed statistically significant findings (P = 0.001, one-tailed test).

**Table 3:** The Z-test results comparing the post-test scores’ means of the flipped classroom and traditional teaching respectively. The one-tailed test yielded a P-value of 0.001; P-value ≤ 0.05 indicates statistical significance; Total marks = 10; Total number of student participants = 100.

Z-Test: Two Samples for Means	Flipped Classroom	Traditional Teaching
Mean	6.543104575	5.944444444
Known Variance	0.749454409	0.603815937
Observations	100	100
Standard deviation	0.865710349	0.777055942
Hypothesized Mean Difference	0	-
z	5.154288182	-
P(Z<=z) one-tail	0.001	-
z Critical one-tail	1.644853627	-
P(Z<=z) two-tail	0.002	-
z Critical two-tail	1.959963985	-

Post-test results of the FC and the TT were categorized into three groups: <5 (unsuccessful), 5-7 (successful), and >7 (excellent) out of 10 marks (Table 4). The categorical comparison of post-test scores between the FC and TT using the Chi-square test (χ2) revealed a statistically significant difference (P = 0.002) among students taught through the FC method (Table 4).

**Table 4:** Categorical comparison of post-test scores using the Chi-square test (χ2). A P-value of ≤ 0.05 indicates statistical significance. FC = flipped classroom; TT = traditional teaching; df = degree of freedom; Total marks = 10; Total number of student participants = 100.

Category	Group FC (n = 100)	Group TT (n = 100)	χ^2^	df	P-value
1 (< 5 marks)	4	10	12.325	2	0.002
2 (5-7 marks)	62	76			
3 (> 7 marks)	34	14			

**Figure 4:**
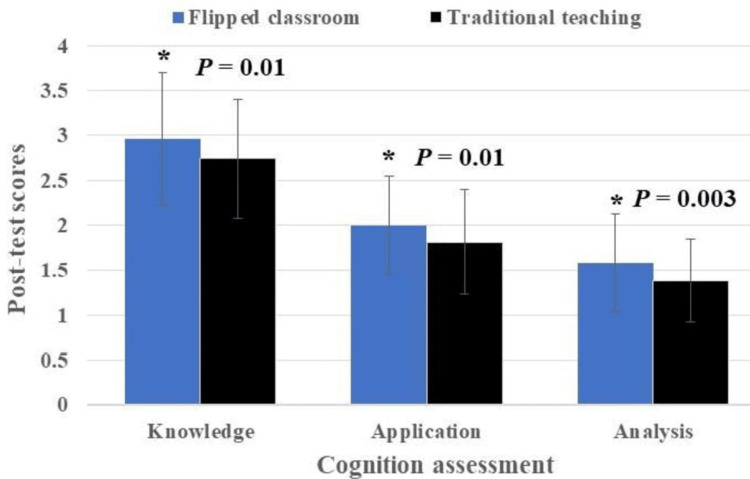
Mean post-test scores for knowledge, apply, and analyze under cognition category were compared between the flipped classroom and traditional teaching techniques using 2D clustered columns. Error bars show standard deviation; * Indicates statistical significance (P ≤ 0.05); Total marks = 10; Total number of student participants = 100.

In 2022, Larsen et al. proposed a revised edition of Bloom's taxonomy, altering the level names and their sequence to: Remember, Understand, Apply, Analyze, Evaluate, and Create [15]. The categories of "remember" and "understand" have been combined into the knowledge category [16]. We have segregated the multiple-choice questions into three categories of cognitive assessment involving knowledge, apply, and analyze components [16]. The set of ten multiple-choice questions was framed into four for knowledge, three for application, and three for analysis. These categories are classified as low level for knowledge, medium level for application, and high level for analysis. Cognitive assessment (involving knowledge, apply, and analyze levels) of mean post-test scores between the FC and TT showed statistical significance in favor of the FC (P = 0.01, P = 0.01, P = 0.003, respectively) (Table 5, Figure 4).

**Table 5:** Mean post-test scores for knowledge, apply, and analyze (cognition assessment) were compared between the flipped classroom and traditional teaching methods. P-value ≤ 0.05 indicates statistical significance; SD denotes standard deviation; Total marks = 10; Total number of student participants = 100.

Cognition assessment	Flipped Classroom Post-test score (Mean ± SD)	Traditional Teaching Post-test score (Mean ± SD)	P-value
Knowledge	2.961 ± 0.736	2.739 ± 0.660	0.01
Apply	2.000 ± 0.542	1.816 ± 0.579	0.01
Analyze	1.583 ± 0.548	1.383 ± 0.464	0.003

**Figure 5:**
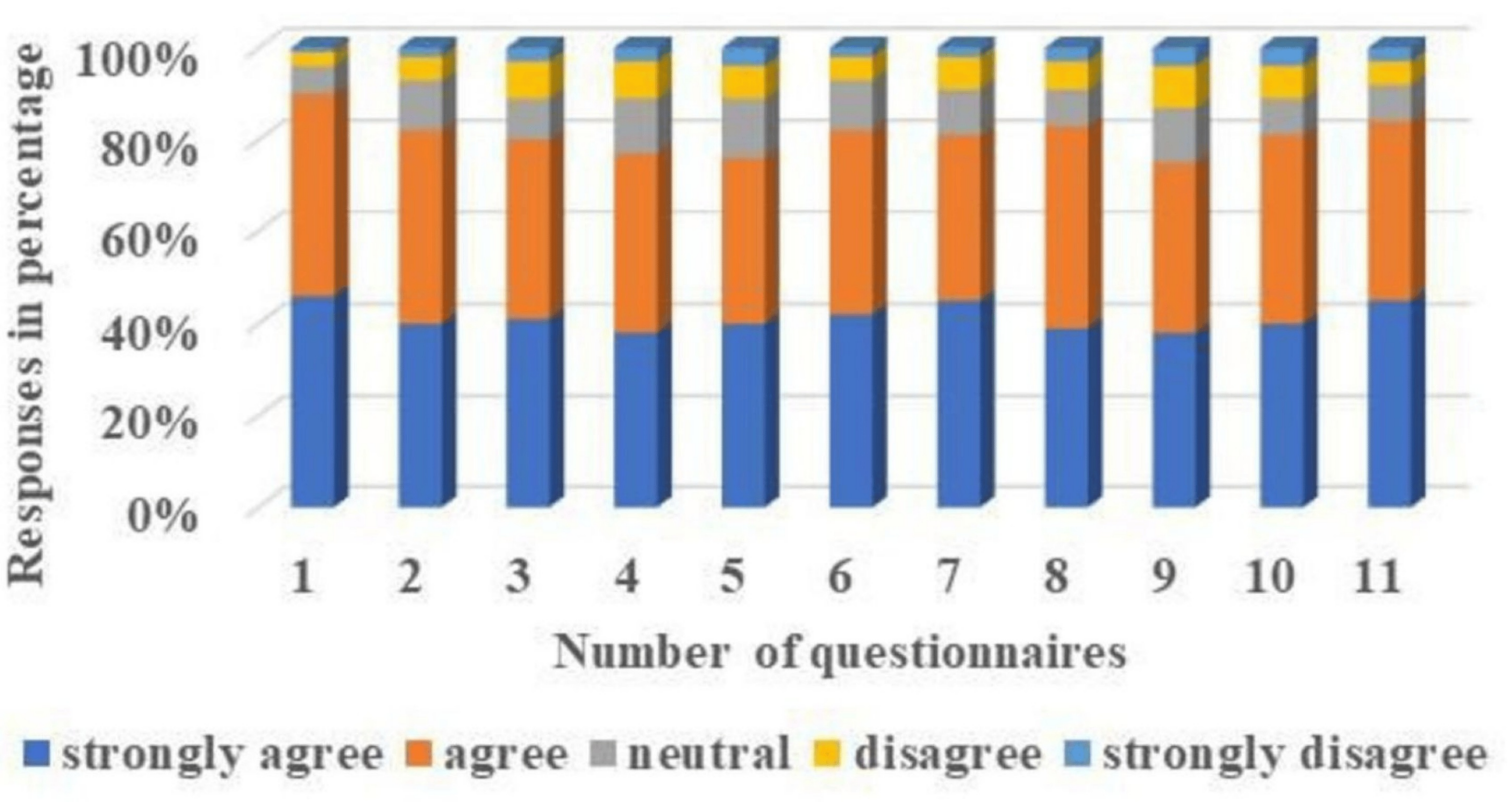
The students’ responses to the questionnaires are based on the Likert Scale, presented using 3D stacked columns. Total number of student participants = 100.

**Figure 6:**
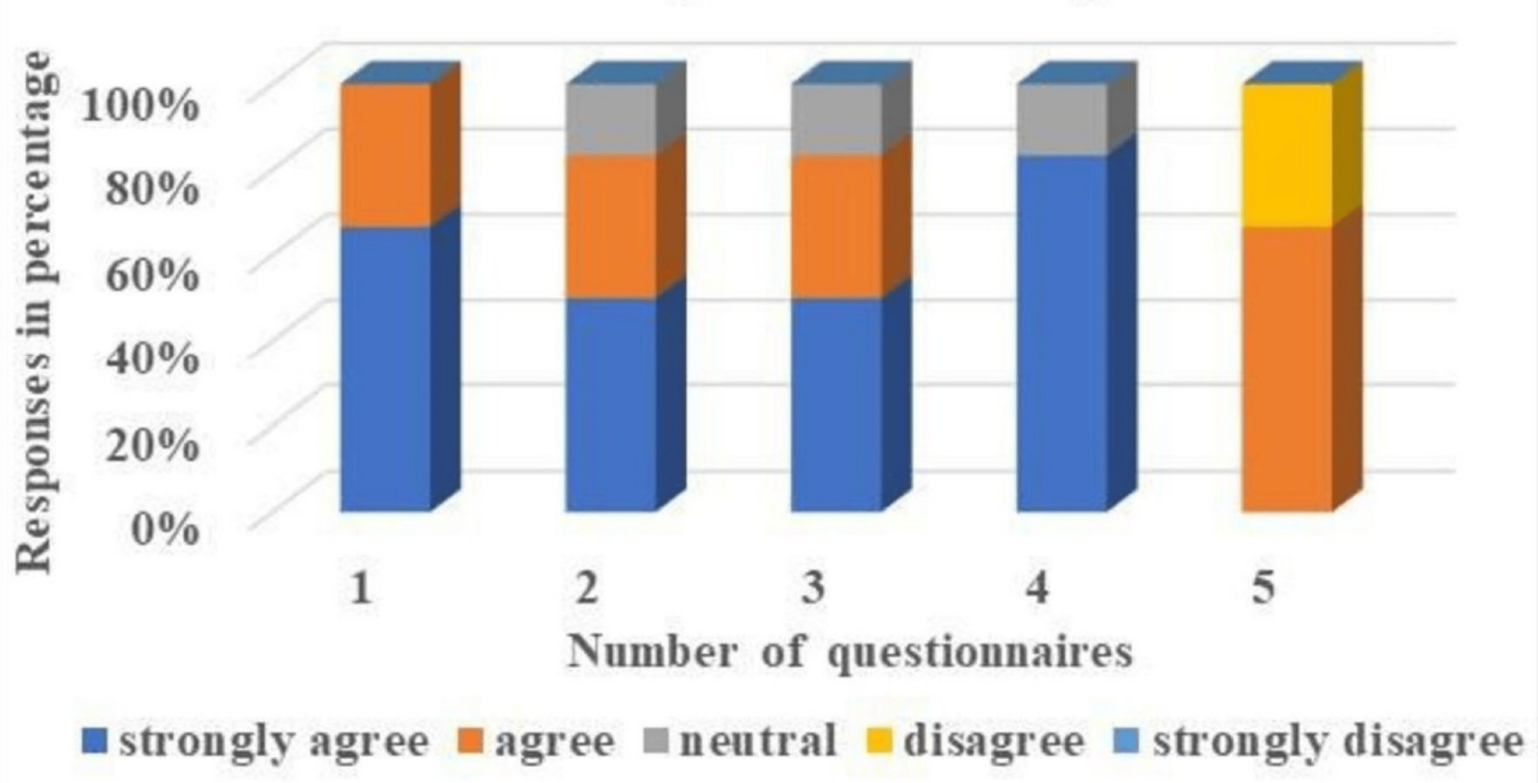
The responses to the questionnaires from the other faculty members of the Anatomy Department were based on the Likert Scale and presented using 3D stacked columns. Number of faculty members = 04.

The details of the feedback response of the students and the faculty, based on the Likert scale, reflecting their perceptions towards the FC, are illustrated in Table 6, Figure 5, and Table 7, Figure 6, respectively.

**Table 6:** Students' responses to questionnaires based on the Likert Scale. Total number of student participants = 100.

S. No	Items	Strongly agree	Agree	Neutral	Disagree	Strongly disagree
1	The flipped classroom is a new model of learning embryology in competency-based medical education	46%	44%	6%	3%	1%
2	The flipped classroom promotes learning motivation in embryology within a competency-based medical curriculum	40%	42%	11%	5%	2%
3	Pre-exposure to classroom study materials enhances understanding and confidence in the embryology topics within a competency-based medical education framework	41%	39%	9%	8%	3%
4	The flipped classroom fosters self-directed study in competency-based embryology topics	38%	39%	12%	8%	3%
5	The flipped classroom method increases active involvement during embryology classes	40%	36%	13%	7%	4%
6	The flipped classroom methods, supplemented by post-test assessments, are beneficial for learning embryology topics in a competency-based medical ecosystem	42%	40%	11%	5%	2%
7	The flipped classroom method generates more interactive and fruitful embryology sessions compared to traditional teaching methods in competency-based medical education	45%	36%	10%	7%	2%
8	The flipped classroom method improves students' ability to approach problem-based questions in embryology topics	39%	44%	8%	6%	3%
9	The flipped classroom model enhances student-teacher relationships within competency-based embryology sessions	38%	37%	12%	9%	4%
10	The flipped classroom methods enhance communication skills among students within an embryology competency-based medical ecosystem	40%	41%	8%	7%	4%
11	The flipped classroom model is an effective way to achieve learning outcomes in competency-based embryology sessions	45%	39%	8%	5%	3%
Average		41.27%	39.73%	9.82%	6.36%	2.82%

**Table 7:** Faculties' responses to questionnaires based on the Likert Scale. Number of faculty participants = 04.

S. No.	Items	Strongly agree	Agree	Neutral	Disagree	Strongly disagree
1	The flipped classroom model effectively addresses all domains of learning required for competency-based medical education in embryology	66.67%	33.33%	0%	0%	0%
2	The flipped classroom model changes the traditional role of teachers in competency-based medical education for embryology	50%	33.33%	16.67%	0%	0%
3	The flipped classroom methods fill the communication gap between teacher and student within a competency-based medical education framework in embryology	50%	33.33%	16.67%	0%	0%
4	The flipped classroom model fosters teamwork in competency-based embryology sessions	83.33%	0%	16.67%	0%	0%
5	The flipped classroom model is more time-consuming and exhaustive compared to traditional teaching methods during competency-based embryology sessions	0%	66.67%	0%	33.33%	0%
Average		50%	33.33%	10.00%	6.67%	0%

Approximately 80% of students acknowledged (41% strongly agree, 39% agree) that the study materials provided before classes improved their comprehension and increased their confidence, while 11% of students differed (8% disagreeing, 3% strongly disagreeing). The majority of students (83%) acquiesced (39% strongly agree and 44% agree) that the FC model helped them attain higher levels of learning objectives, while 9% of students forbade this notion (6% disagreeing, and 3% strongly disagreeing). About 81% of students supported (41.27% strongly agree, and 39.73% agree) the adoption of the FC method, and 9.18% of students did not favor it (6.36% disagree, and 2.82% strongly disagree) (Table 6). All the faculty members (66.67% strongly agreed and 33.33% agreed) expressed their approval of the FC as the optimal teaching and learning tool to effectively address all necessary domains in the current context of competency-based medical education. Maximum faculty members overwhelmingly acknowledged that the implementation of the flipped classroom (FC) has significantly transformed the traditional role of teachers. This transformation has rendered them more dynamic and proficient in guiding, facilitating, and mentoring students. The approval rates demonstrate substantial support, with 50% strongly endorsing this transformation and 33.33% indicating agreement. Our research findings reveal that a substantial majority of 66.67% of faculty members acknowledge that the implementation of the FC model involves a significant amount of time and demands.

This suggests that they perceive it to require more effort and resources compared to traditional teaching methods. However, it is important to note that the remaining 33.33% of faculty members express their disagreement with this viewpoint. This indicates that there is diversity in opinions among faculty members regarding the time consumption and demands associated with the FC model (Table 7).

## Discussion

The purpose of the present research investigation is to evaluate the effectiveness of using the FC model as opposed to TT methods in enhancing the comprehension of embryology among first-year MBBS students. Our goal is to empower our students, enabling them to emerge as competent medical graduates equipped with the necessary knowledge, skills, attitude, and communication competence required to excel in the Indian healthcare landscape as well as globally. In doing so, they will have the ability to positively impact the health and well-being of individuals and communities worldwide.

The findings of the current study demonstrate the effectiveness of the FC model in enhancing understanding of embryology, fostering knowledge acquisition, promoting the application of knowledge, and developing critical analysis skills among students compared to the TT method (Tables 3-5). The study conducted by Jha et al. provides valuable insights into the effectiveness of the flipped classroom (FC) model on various aspects of students' performance. The results indicate that, although there were no significant differences in knowledge and application scores between FC and TT students, those in the FC group outperformed their TT counterparts on analysis-related questions [16]. In a previous study, the FC model was shown to improve semester grades for low-performing students compared to the TT method. Additionally, students in the FC group demonstrated greater accuracy in answering questions that required higher-order thinking skills, such as applying and analyzing concepts, on the final examination [17].

Feedback surveys involving both students and faculty members in the anatomy department were conducted to gather their perspectives on the FC approach. The feedback indicated that the flipped classroom model was effective in enhancing the students' competency in embryology. They also found the sessions conducted using the FC method highly beneficial and stated that it helped them achieve higher levels of learning objectives. Faculty members acknowledged that the implementation of the FC model significantly transformed their role, making them more dynamic and proficient in guiding, facilitating, and mentoring students [3]. While some faculty members expressed concerns about the time commitment associated with the flipped classroom model, the majority rejected this notion, emphasizing the significant advantages it offers. Additionally, both students and faculty members expressed positive perceptions of integrating the FC model into the curriculum (Table 6, Table 7).

The mechanism of early embryological development of human beings is discussed under the embryological curriculum of Anatomy. The embryology topics have always remained challenging for learners to understand, comprehend, and apply in problem-based learning [10,11]. It becomes a tough task for the 1st year MBBS students to build up a crystal-clear concept about prenatal embryological concepts with the help of the traditional teaching-learning method within the stipulated academic schedule. This is the critical factor that a larger student section prefers to remain aloof from the embryology section. Later on, when these students go for clinical postings, they find it very difficult to analyze the clinical cases that have an embryological basis, as they lack the competency relevant to the lower order of Bloom’s taxonomy due to the failure to achieve that during the 1st year of Anatomy classes of human embryology. Studying human embryology is very important because it provides essential knowledge to comprehend different clinical issues and manage them more efficiently [10,11]. That helps to build up proper healthcare strategies for better reproductive outcomes, which is a great boon for society.

In the present study, we have chosen the FC and the embryology topics because of their relevance in the medical curriculum. Currently, the field of medical science is undergoing rapid evolution [1,7,10,11]. The limitations imposed by TT methodology pose formidable challenges for medical students striving to achieve a comprehensive understanding of subjects within a confined timeframe [1,7,17]. In the contemporary era, as medical professionals worldwide increasingly adopt competency-based medical education, medical students face significant challenges in attaining the desired learning objectives across various competency domains [1,3,5]. Embracing technological progress enables the revolutionary transformation of current medical education through the integration of advanced technology [6,7,10,12,13]. This transition has the potential to enhance the productivity and engagement of learning, ultimately fostering a comprehensive understanding critical for producing competent medical professionals globally, capable of effectively managing healthcare systems. We require an engaging, dynamic, efficient, and technologically friendly approach that empowers students to reach all cognitive levels outlined in Bloom’s taxonomy. The FC stands out as an emerging teaching-learning tool that has the potential to be more productive than the TT in the current landscape of medical education [3,7,16,17]. The FC technique is more kinetic, focused, and collaborative than TC in the field of medical education and training. The FC increases self-confidence, grades, interest, activity in the class, interaction, satisfaction, and longevity, and increases overall performance in anatomy [3].

Unlike the TT, which primarily emphasizes lower levels of cognitive work, such as recalling facts and understanding basic concepts according to Bloom’s revised taxonomy, the FC model encourages students to participate in higher levels of cognitive skills [2,3,6,16,17]. Overall, this study's findings have a significant scientific impact, highlighting the benefits of the flipped classroom model in improving embryology understanding and providing a foundation for further research and development in medical education.

Limitation

It is worth noting that this study was conducted with one hundred first-year MBBS students, and further research with a larger number of sample size is needed to explore the applicability and effectiveness of the flipped classroom model across different tiers of medical education. Additionally, future studies should also investigate potential challenges and barriers to implementation, as well as strategies for optimizing the flipped classroom model in medical education.

## Conclusions

The flipped classroom model stands out as an emerging teaching-learning tool that has the potential to be more productive than traditional teaching in the current landscape of medical education. Our study provides strong evidence that the flipped classroom model is effective in improving embryology understanding among first-year MBBS students. It not only enhances knowledge acquisition but also promotes higher-level cognitive skills and critical thinking. The positive feedback from students and faculty members further supports the integration of this model into the medical curriculum. These findings have important implications for educational practice, can lead to improved learning outcomes, and better prepare students for future challenges in their academic and professional careers. However, further research is necessary to address potential challenges and optimize the implementation of the flipped classroom model in medical education. It is worth noting that this study was conducted with first-year MBBS students, and further research is needed to explore the applicability and effectiveness of the flipped classroom model across different tiers of medical education. Additionally, future studies should also investigate potential challenges and barriers to implementation, as well as strategies for optimizing the flipped classroom model in medical education.

## Appendices

Appendix 1

**Table 8:** Students’ questionnaires

Students’ questionnaires

S. No.	Items	Strongly agree	Agree	Neutral	Disagree	Strongly disagree
1	The flipped classroom is a new model of learning embryology in competency-based medical education					
2	The flipped classroom promotes learning motivation in embryology within a competency-based medical curriculum					
3	Pre-exposure to classroom study materials enhances understanding and confidence in the embryology topics within a competency-based medical education framework					
4	The flipped classroom fosters self-directed study in competency-based embryology topics					
5	The flipped classroom method increases active involvement during embryology classes					
6	The flipped classroom methods, supplemented by post-test assessments, are beneficial for learning embryology topics in a competency-based medical ecosystem					
7	The flipped classroom method generates more interactive and fruitful embryology sessions compared to traditional teaching methods in competency-based medical education					
8	The flipped classroom method improves students' ability to approach problem-based questions in embryology topics					
9	The flipped classroom model enhances student-teacher relationships within competency-based embryology sessions					
10	The flipped classroom methods enhance communication skills among students within an embryology competency-based medical ecosystem					
11	The flipped classroom model is an effective way to achieve learning outcomes in competency-based embryology sessions					

Appendix 2

**Table 9:** Faculties’ questionnaires

Faculties’ questionnaires

S. No.	Items	Strongly agree	Agree	Neutral	Disagree	Strongly disagree
1	The flipped classroom model effectively addresses all domains of learning required for competency-based medical education in embryology					
2	The flipped classroom model changes the traditional role of teachers in competency-based medical education for embryology					
3	The flipped classroom methods fill the communication gap between teacher and student within a competency-based medical education framework in embryology					
4	The flipped classroom model fosters teamwork in competency-based embryology sessions					
5	The flipped classroom model is more time-consuming and exhaustive compared to traditional teaching methods during competency-based embryology sessions					

Appendix 3

**Table 10:** Post-test sample of MCQ delivered through TT

Post-test MCQ sample, Department of Anatomy, All India Institute of Medical Sciences, Raebareli. Topic: Third week of development (Embryology) Traditional teaching method (TT)						
S. NO.	QUESTIONS	OPTION: A	OPTION: B	OPTION: C	OPTION: D	ANSWER
1	Gastrulation begins with the formation of	Yolk sac	Somites	Neural tube	Primitive streak	
2	Which germ layer is formed from the epiblast?	Endoderm	Ectoderm	Mesoderm	All are correct	
3	Which embryonic structure induces the formation of the neural plate?	Endoderm	Hypoblast	Neural crest cells	Notochord	
4	The primitive node is present at which end of the Primitive streak?	Cranial	Caudal	Ventral	Dorsal	
5	The epiblast cells migrating through the more caudal part of the primitive streak give rise to	Notochord	Paraxial mesoderm	Intermediate mesoderm	Lateral plate mesoderm	
6	Invaginating cells coming from the epiblast displace the hypoblast and give rise to the future?	Ectoderm	Mesoderm	Endoderm	Both B & C	
7	A failure in cell migration through the primitive streak during the third week of development may most likely result in which condition?	Absence of mesodermal organs	Hydatidiform mole	Neural tube formation	Situs inversus	
8	Persistence of remnants of the primitive streak in the fetus may give rise to?	Sacrococcygeal teratoma	Anencephaly	Spina bifida	Sirenomelia	
9	What is the likely embryological source of error in disrupted left-right axis formation?	Failure of the cloacal membrane rupture	Defective neural fold fusion	Incomplete somite segmentation	Dysfunctional cilia in the primitive node	
10	Which is the correct statement regarding the developmental role of the notochord?	Provides a proper signal for the development of the axial skeleton	Defines the embryonic axis	It influences gut tube development	All are correct	

Appendix 4

**Table 11:** Post-test sample of MCQ delivered through FC

Post-test MCQ sample, Department of Anatomy, All India Institute of Medical Sciences, Raebareli. Topic: Urinary System (Embryology), Flipped classroom method (FC)						
S. NO.	QUESTIONS	OPTION: A	OPTION: B	OPTION: C	OPTION:D	ANSWER
1	The metanephric kidney develops from	Intermediate mesoderm	Ureteric bud	Metanephric blastema	All are correct	
2	The metanephros appears at	4^th^ week	5^th^ week	7^th^ week	8^th^ week	
3	The urogenital ridge is derived from	Lateral plate mesoderm	Paraxial mesoderm	Intermediate mesoderm	Endoderm	
4	Definitive kidney becomes functional near the	4^th^ week	8^th^ week	12^th^ week	16^th^ week	
5	What will be the likely clinical presentation in a fetus with bilateral renal agenesis?	Oligohydramnios	Polyhydramnios	Normal amniotic fluid	Both A & B	
6	The WT1 gene mutation may hinder the development of	Ureter	Major & Minor calyces	Metanephric mesenchyme	All are correct	
7	Failure of interaction between the ureteric bud and the metanephric blastema will result in	Lobulated kidney	Renal agenesis	Malrotated kidney	Horseshoe kidney	
8	The bifid ureter, a congenital anomaly, arises due to?	Early bifurcation of the ureteric bud	Ureteric bud fails to join the metanephric blastema	Degeneration of the mesonephric duct	Subdivision of the ureteric bud inside the metanephric tissue.	
9	The ascent of the kidney occurs due to?	A decrease in the body curvature	Increase in the body curvature	Growth of the body in the lumbar & sacral region	Both B & C	
10	The formation of the nephron is facilitated by the interaction of the metanephric blastema with?	Ureteric bud	Cloaca	Paramesonephric duct	Mesonephric duct	
